# Association Between Patient Sociodemographic and Clinical Characteristics and Acute Mental Health Service Utilization Within One Year Following Enrollment in the Rapid Access and Stabilization Program in Nova Scotia

**DOI:** 10.3390/jcm14155241

**Published:** 2025-07-24

**Authors:** Medard K. Adu, Samuel Obeng Nkrumah, Belinda Agyapong, Gloria Obuobi-Donkor, Ejemai Eboreime, Lori Wozney, Vincent Israel Opoku Agyapong

**Affiliations:** 1 Department of Psychiatry, Faculty of Medicine, Dalhousie University, Halifax, NS B3H 2E2, Canada; md269491@dal.ca (M.K.A.); sm416395@dal.ca (S.O.N.); bagyapon@ualberta.ca (B.A.); gloria.ob@dal.ca (G.O.-D.); ejemai.eboreime@dal.ca (E.E.);; 2 Mental Health and Addictions Program, Nova Scotia Health, Halifax, NS B3S 0H6, Canada

**Keywords:** rapid access, acute care utilization, mental health, emergency department, inpatient admission, mobile crisis calls

## Abstract

**Background/Objectives**: The Rapid Access and Stabilization Program (RASP), launched in Nova Scotia in April 2023, aims to improve timely psychiatric care, reduce reliance on emergency services, and provide early intervention. This study describes the sociodemographic and clinical characteristics of the RASP participants and examines their association with acute service use. **Methods**: This cross-sectional descriptive study used self-reported surveys and administrative data from 738 RASP participants. Descriptive statistics summarized key sociodemographic and clinical variables. Associations between these characteristics and acute service use (emergency department visits, inpatient admissions, and mobile crisis calls) were examined using chi-square and Fisher’s Exact tests. Bonferroni correction was applied for multiple comparisons. **Results**: The sample was predominantly female (65.2%) and aged 20–40 years (38.4%). Despite high rates of severe anxiety (53.9%) and depression (36.0%), acute service use was low: emergency department visits (7.2%), mobile crisis calls (1.0%), and inpatient admissions (0.8%). Preliminary analyses showed that education level and housing status were associated with ED visits and inpatient admissions. However, these associations did not remain statistically significant after Bonferroni correction. **Conclusions**: Although mental health symptom severity was high, acute mental health service use remained low after RASP enrollment, indicating the program’s potential in reducing reliance on crisis services. No participant characteristics were significantly associated with acute service use after adjustment, underscoring the complexity of predicting utilization and the need for robust multivariable models. Continued investment in rapid access programs may be essential to improving timely mental health care and supporting early intervention strategies.

## 1. Introduction

### 1.1. Mental Health Service Access in Canada and Nova Scotia

Mental health disorders remain a major public health concern in Canada, with one in five Canadians experiencing conditions such as anxiety, depression, or substance use each year [1]. According to Statistics Canada, nearly 18% of Canadians, approximately 5.3 million people, reported a need for mental health support. However, only 56.2% indicated that their needs were fully met, while the remaining 43.8% had partially met or unmet needs, particularly among those without a regular healthcare provider [1,2]. Despite this high prevalence, nearly half of those who sought mental health care reported that the existing system did not fully meet their needs, often citing barriers such as cost and lack of information [1,3]. Common barriers to accessing care include not knowing where to seek help, long wait times, stigma, and inability to pay for services [2]. The economic toll of inadequate mental health care is substantial as well, and the overall yearly economic burden of mental illness in Canada is estimated to exceed CAD 50 billion when accounting for health care costs, lost productivity, and social services [4]. This combination of high prevalence and high unmet needs underscores the importance of improving access to mental health services provincially and nationally.

Provincially, Nova Scotia has similar trends to the national challenges in mental health service access [5]. Similar proportions of Nova Scotians report unmet psychological needs, indicating a considerable “mental health care gap” in the province [5]. In Nova Scotia, the most commonly reported unmet needs revolve around difficulty accessing counseling services and the financial costs of care [5]. These gaps are consistent with the broader Canadian context and point to systemic issues such as provider shortages and funding limitations. Long wait times for outpatient mental health services have been a particular concern in Nova Scotia, as delays in seeing specialists or therapists can lead individuals to become more acute or to seek help from emergency departments (EDs) out of desperation [6,7]. Indeed, many individuals end up using the ED as their first point of contact for mental health care when timely outpatient care is not available [6]. This pattern is viewed as an indicator of poor access to routine mental health services and puts additional strain on emergency and inpatient systems. Thus, there has been a provincial push to close this mental health treatment gap by innovating how mental health services are delivered, aiming to provide help earlier by increasing direct access to psychiatric consultation and more efficiently to those in need at the right time.

### 1.2. Rationale for Rapid Access Mental Health Programs

To address the above disparities in the mental health care system, stakeholders and health authorities have resorted to rapid access models of care to improve access to services and reduce pressure on acute care centers. Early intervention in mental health care has well-documented significance; thus, it can prevent the worsening of symptoms, lead to rapid recovery outcomes for service users and their families, improve quality of life, and reduce long-term disability [5,8]. By seeking early interventions for mental health problems before they reach a crisis stage, these prompt and rapid access interventions can also help reduce the overreliance on high-cost services like ED visits and hospital admissions [5,8,9]. Therefore, there is a strong need to integrate short-term mental health interventions that are easily accessible and cost-effective into the continuum of care. These rapid access programs align with the stepped-care models of care, in which patients receive care tailored to their specific needs [5]. The stepped-care model ensures that moderate issues are managed in the community before they become severe enough to require acute services such as hospitalization, thereby improving system efficiency and patient experience.

Within this context, Nova Scotia Health and the Department of Psychiatry at Dalhousie University launched the Rapid Access and Stabilization Program (RASP) in April 2023 to address this decades-old treatment gap in access and to provide timely mental health support to individuals in need [7]. The RASP aims explicitly to increase direct access and reduce long wait times for psychiatric consultation, decrease utilization of emergency services, and provide early intervention for individuals with mental health concerns. The program operates as a bridge between primary care and specialized mental health services. Primary healthcare providers can refer patients directly to RASP psychiatrists, bypassing the usual lengthy referral protocols to central assessment and community mental health centers [5]. Psychiatrists and mental health staff in the RASP clinic work on a rotating schedule to provide rapid assessments, diagnostic clarification, and short-term stabilization plans for referred clients. After evaluation, patients are reconnected to their primary care providers with comprehensive consultation reports to guide ongoing management and ensure continuity of care. This collaborative model enables timely specialist input while preventing unnecessary follow-ups and empowering primary care providers. RASP’s primary focus is rapid stabilization of emerging or worsening mental health problems through timely medication initiation or adjustment, treatment planning, and linkage to community resources. By promoting early detection, prompt intervention, and clear care plans, the program aims to reduce reliance on emergency departments and inpatient units that would otherwise manage these cases during crises [5].

Preliminary findings suggest that the Nova Scotia RASP has been effective in significantly increasing direct access to psychiatric consultation within the region in its first year of operation. Thus, the Rapid Assessment Clinic has shortened wait times for care, which is associated with improved patient satisfaction and outcomes in its early stages [7]. By offering access to direct psychiatric consultation within days or weeks, rather than the usual months to years, the rapid access program facilitates the early detection and provision of necessary support, thereby preventing deterioration in patient conditions. Furthermore, moving clients away from the ED unless critically needed helps prevent the adverse experiences associated with emergency care. As part of a broader system transformation, RASP demonstrates how reallocating resources to rapid access and stabilization centers can alleviate downstream pressures on traditional mental health care systems [7]. In summary, the rationale for RASP and other rapid access and early intervention initiatives is grounded in the dual goals of improving patient outcomes through prompt, accessible care and improving system efficiency by reducing avoidable acute care service utilization.

### 1.3. Known Determinants of Acute Mental Health Service Utilization

Despite the ongoing advancement in access to mental health services through targeted innovative intervention across the globe, specific individuals will still require the services and use of acute mental health services such as hospitalization, ED visits, and mobile crisis interventions. Understanding the factors that predispose patients to high acute mental health utilization is crucial for targeted preventive measures aimed at mitigating this long-standing health problem [10]. Identifying these variables that influence the frequency of acute mental health service use is of prime importance to understanding and prevention of this phenomenon, which mainly results in a worsening prognosis and poor outcomes in terms of course, quality of life, well-being, and effective functioning of the affected individuals [11,12,13].

Previous studies have consistently identified a variety of risk factors, including sociodemographic and socioeconomic disadvantage, housing status, substance use disorders, clinical characteristics (severe psychiatric diagnoses), recurrent acute mental health services use, and gaps in outpatient care continuity [14,15,16]. Demographic characteristics, such as age, sex, and ethnicity, may impact service use patterns, although the findings are mixed [16,17]. Younger individuals and marginalized groups often rely more heavily on emergency care due to barriers to accessing regular mental health services [17]. Importantly, many high users are already under psychiatric care but continue to experience crises due to complex, unmet needs [18,19,20]. Protective factors, such as employment and strong social support networks, can mitigate risk [21,22].

These insights highlight the significance of early interventions and rapid access initiatives like Nova Scotia’s Rapid Access and Stabilization Program, which aim to address these upstream predisposing factors by providing prompt and timely intervention, coordinated care support systems, and connecting individuals who require help to appropriate community mental health resources that potentially reduce acute mental health service utilization. Given the program’s design to provide early stabilization, we anticipated low post-intake acute service use, with limited power to detect small to moderate associations due to expected low event rates. These considerations informed the descriptive nature of our analysis.

### 1.4. Theoretical Framework: Andersen’s Behavioral Model of Health Services Use

This study is based on Andersen’s Behavioral Model of Health Services Use, a commonly employed framework for analyzing factors that affect healthcare utilization. Originating in 1968 and evolving over the years, the model categorizes the determinants into three categories: predisposing characteristics (such as age, gender, and education), enabling resources (including income, housing, and access to care), and the need for care (encompassing perceived or clinically evaluated symptom severity) [23]. Andersen’s model is particularly applicable in mental health research, where individual and systemic factors shape access to care [24,25]. In RASP, the model provides a framework for analyzing how participant traits influence their use of urgent mental health services, including emergency visits, inpatient stays, and mobile crisis calls.

This study utilizes the model to analyze intake data and linked administrative records, exploring the relationships between patient characteristics and the use of acute services within one year post-enrollment. It examines predisposing factors, such as age and education, enabling factors, including income and housing, and need factors assessed through clinical scales (GAD-7, PHQ-9, and WHO-5). Grounding the analysis in Andersen’s model, the study provides a comprehensive, theory-based understanding of how services are utilized following brief psychiatric consultations, highlighting ways to enhance access, equity, and system efficiency.

### 1.5. Study Aim

This study aims to describe the sociodemographic and clinical characteristics of individuals who accessed RASP between April 2023 and April 2024, and to examine how these traits are associated with the use of acute mental health services such as ED visits, psychiatric admissions, and mobile crisis calls, within one year after enrollment. Using Andersen’s model, this analysis systematically explores whether and how predisposing factors (such as age, education, and gender), enabling resources (such as income, housing status, and employment), and assessed needs (including symptom severity and well-being scores) influence different patterns of emergency service utilization. The study employs both self-reported intake assessments and linked provincial mental health administrative data to identify combinations of factors that may increase the risk of post-intake acute care use among RASP participants. This approach, aligned with Andersen’s model, provides a straightforward interpretation of the results, indicating whether clinical needs, socioeconomic factors, or predisposing demographics have a greater influence on service use.

By doing so, the study not only deepens the theoretical understanding of access and utilization in mental health systems but also offers practical guidance for improving the RASP model. In particular, it encourages data-driven refinement of triage processes, focuses on high-risk groups, and promotes early interventions to reduce dependence on expensive acute care services. Ultimately, by basing the analysis on Andersen’s Behavioral Model, the study provides a clear conceptual framework for evaluating service use outcomes and improves the significance of its results for mental health policy, program planning, and resource allocation.

## 2. Method

### 2.1. Study Setting

This study took place in the central zone of Nova Scotia. Mental health care in Nova Scotia is publicly funded and delivered by the Nova Scotia Health Authority through the Mental Health and Addictions Program, which serves residents across four administrative zones: Central, Eastern, Western, and Northern. RASP, a Tier 3 model of care, was established and implemented in April 2023 and is located at the QE II Health Sciences Centre in Halifax, the provincial capital. RASP explicitly serves patients within the central zone (CZ)

### 2.2. Study Design

This descriptive and observational study aims to identify the key sociodemographic and clinical factors associated with the frequency of acute mental health service utilization one year after assessment in individuals who accessed mental health care within the RASP between April 2023 and April 2024. In this case, acute mental health services referred to inpatient admissions, emergency department visits, and mobile crisis calls made by participants post-RASP assessment.

### 2.3. Data Collection and Study Participants

The study participants included all patients who attended RASP and consented to have their health service utilization records, including ED visits, mobile crisis calls, and inpatient admissions, extracted from the provincial information systems during the study period. Key sociodemographic and clinical profiles were recorded for each participant during the pre-assessment self-assessment forms, completed 30 min before the patient saw their psychiatrist in the RASP clinic. This covered the following areas: sociodemographic characteristics (age, sex at birth, gender, relationship status, educational levels, employment and housing status, income range, and ethnicity). At the same time, the clinical profiles included variables such as known psychiatric diagnoses (Anxiety, major depressive disorder, and the well-being index). Furthermore, participants’ health utilization information, including ED visits, mobile crisis calls, and inpatient admissions, was extracted from provincial information systems one year after their assessment at RASP. The data collection window spanned from 1 April 2023, to 30 April 2024.

### 2.4. Missing Data Management

All participants completed standardized intake forms during enrollment, resulting in a complete dataset. For analysis, any cases with missing data on relevant variables were excluded through listwise deletion. This method was selected because missing data was minimal (under 5%) and the study was primarily descriptive. Listwise deletion helps maintain consistency across comparisons and is suitable when data are presumed to be missing completely at random (MCAR).

### 2.5. Outcome Measures

The outcome measures were the three binary indicators of acute mental health service utilization one year post-RASP assessment (inpatient admissions, emergency department visits, and mobile crisis call), focusing on identifying the associations between participants’ sociodemographic and clinical characteristics and their acute mental health services.

### 2.6. Data Analysis

All statistical analyses were conducted using the Statistical Package for Social Sciences (SPSS) version 29 for Windows. The statistical significance threshold was *p* < 0.05 (two-tailed). The study used descriptive statistics to summarize participants’ sociodemographic and clinical profiles. Differences in post-RASP assessment acute mental health service utilization (emergency department visits, inpatient admissions, and mobile crisis calls) were assessed using chi-square (χ^2^) tests or Fisher’s Exact Test where the expected cell counts were smaller than expected. Phi coefficient or Cramer’s was used to quantify effect sizes for the categorical associations, depending on the number of categories within each variable. Using these metrics allowed for the examination of the strength of associations, with a cut-off point of 0.1 (small), 0.3 (medium), and 0.5 (large). To account for multiple comparisons, a Bonferroni correction was applied. Although conservative, this method was chosen to control the family-wise error rate and minimize false-positive findings rigorously. Given the limited number of hypotheses tested, the Bonferroni method was preferred over less stringent alternatives, such as the False Discovery Rate (FDR).

### 2.7. Ethical Considerations

This study was conducted following the Declaration of Helsinki, and ethical approval was obtained from the Nova Scotia Health Research Ethics Board (REB File #1028254). All RASP patients signed an informed consent form as part of their pre-assessment package, which allowed investigators to access their health services utilization records upon signing.

## 3. Results

### Participant Characteristics by Gender

As shown in Table 1, the analysis included 738 participants. Of these, most respondents, 281 (38.4%), were between 20 and 40 years old, and 477 (65.2%) identified as female. The majority of participants, regardless of gender, identified as Caucasian, with 642 (89.0%) reporting this ethnicity. Employment status indicated that 409 (56.3%) were employed, with males at 145 (57.3%) and females at 260 (56.0%), showing similar rates. Income levels varied, with the largest group, 210 (30.6%), earning between CAD 29,592 (CAN) and CAD 59,180 (CAN). Regarding relationship status, most participants were married or in a partnership, accounting for 369 (50.5%). Educational attainment was generally high, with 444 (60.8%) reporting post-secondary education. Notably, males were more likely than females to report post-secondary trade qualifications (11.1% versus 7.1%). Most participants lived in rented accommodation (298, 40.8%) or owned their homes (37.8%).

Regarding clinical indicators, anxiety severity measured by GAD-7 showed that 393 (53.9%) of participants reported severe anxiety, with females having a higher rate (57.5%) than males (47.4%). Similarly, for major depressive symptoms (PHQ-9), 357 (48.8%) reported moderate depression, while 263 (36.0%) had severe major depressive disorder (MDD), with females showing higher severity (39.4%) compared to males (29.5%). Conversely, scores on the WHO-5 Well-being Index indicated that 660 (90.2%) of all participants reported high well-being, with males (86.6%) being slightly less likely to report high well-being compared to females (91.9%)

Furthermore, the twelve-month post-RASP assessment revealed that only 7 (1.0%) participants made a mobile crisis call, 53 (7.2%) made visits to the emergency department, and 6 (0.8%) were admitted to inpatient psychiatric units post-RASP assessment. Although overall acute mental health service utilization was low across all genders, male participants had slightly higher emergency department visit rates (7.1%) and inpatient admission rates (1.6%) compared to their female colleagues (7.5% and 0.4%, respectively).

A summary of the results of the chi-square/Fisher’s Exact analysis showing the sociodemographic and clinical variables associated with acute mental health services utilization of RASP participants one year post-assessment is displayed in Table 2.

The bivariate analyses revealed no statistically significant associations between participants’ age category, gender, or sex at birth and any of the three acute mental health services utilization outcomes. However, the analysis detected some trends. For example, participants aged <25 reported slightly higher proportions of emergency department visits post-assessment than the older groups, though no statistically significant associations were observed (χ^2^ = 3.95, *p* = 0.23). Likewise, no statistically significant relationship was found between participants’ ethnicity, employment status, relationship status, income levels, and acute mental health service utilization, with *p*-values consistently greater than 0.05.

However, educational level and housing status revealed statistically significant associations in certain outcome domains. Specifically, educational attainment was statistically significantly associated with both emergency department visits (χ^2^ = 9.11, *p* = 0.04, Cramer’s V = 0.12) and inpatient admissions (χ^2^ = 10.79, *p* = 0.02, Cramer’s V = 0.14) following the RASP assessment. Similarly, participants’ housing status showed a statistically significant relationship with inpatient admission (χ^2^ = 10.32, *p* = 0.01, Cramer’s V = 0.19) post-assessment, while those in non-traditional or unstable housing displayed higher rates.

Regarding mental health symptom severity (anxiety and depression) presented by participants and measured by GAD-7 and PHQ-9, respectively, they were not statistically significantly associated with any of the acute mental health service outcomes under study, with *p*-values consistently >0.05. However, descriptive analysis showed increased ED visits among those with severe anxiety or depression. Likewise, the psychological well-being (WHO-5 scores) was not significantly associated with post-assessment service use, though descriptive analysis showed increased ED visits among those with severe anxiety or depression.

Notwithstanding the above results, a Bonferroni correction was applied to adjust the significance threshold and account for multiple comparisons, reducing the likelihood of false positives (Type I errors). With the adjusted alpha set at *p* < 0.004, none of the sociodemographic and clinical variables tested for association with post-assessment acute mental health service use showed statistically significant associations, including educational level and housing status, which had initially demonstrated significance at *p* < 0.05. This may imply that the observed association between educational attainment and housing status with post-assessment acute mental health services use, specifically ED visits and inpatient admissions, may be due to random chance.

## 4. Discussion

This study presents a comprehensive overview of the sociodemographic and clinical profiles of individuals who accessed mental health services through RASP between April 2023 and April 2024, along with factors associated with acute mental health services usage one year post-RASP assessment. It offers critical insight into the characteristics of the assisted population and how their demographic, social, and clinical characteristics shape the trajectories of acute mental health services utilization.

The findings revealed that most participants were females between 20 and 40 years old. This result is consistent with previous studies that suggest that females are more likely than males to seek help for mental health concerns [26,27]. That early adulthood is deemed to represent a critical period where the onset of many mental health problems begins [28,29]. Furthermore, a great majority of study participants reported high educational attainment while being employed. This finding suggests that participants had relatively stable socioeconomic status, although a sizable proportion still reported belonging to the lower income brackets. Consistent with previous mental health studies, these findings highlight the reality that even with higher educational attainment and employment, socioeconomic disadvantages may persist [30,31].

Regarding the baseline clinical variables, the findings revealed a greater burden of mental health issues among the study participants. Thus, more than half reported severe anxiety symptoms on the self-assessed anxiety measure (GAD-7), while about a third of them reported meeting the criteria for MDD as measured on the PHQ-9 scale. Notably, females reported higher levels of both depression and anxiety symptoms. This finding is consistent with a broader epidemiological trend suggesting an increased prevalence of mood disorders in women [32,33,34]. Interestingly, despite the high rate of mental health symptoms reported, over 90% of study participants indicated having a high quality of life as measured by the WHO-5 Well-being Index. Consistent with previous studies, the difference between clinical symptoms and subjective well-being may reflect the influence of protective factors and the resilience of participants, due to other social factors not captured in our dataset [35,36,37].

Again, regardless of the high rates of clinical symptoms reported by the study participants, acute mental health usage remained low, with only about 7.2% who accessed ED visits, 0.8% who got admitted, and 1.0% who made mobile calls one year post-RASP enrollment. Although the observed effect sizes (Cramer’s V = 0.12–0.19) were statistically small, they may still hold practical significance in population mental health contexts, where modest associations can inform service planning and policy at scale. Although causal inference cannot be drawn from our observational design, consistent with the previous literature, the low rates of acute mental health service utilization observed in this study may reflect the potential benefits of the Rapid Access programs. This aligns with existing evidence that early, integrated mental health interventions can reduce reliance on high-cost acute care services [38,39,40].

### 4.1. Sociodemographic and Clinical Factors Associated with Acute Mental Health Services Use

This study did not find statistically significant associations between the sociodemographic variables (age, sex, ethnicity, employment, income, relationship status, and clinical symptom severity variables) and acute mental health services utilization. This finding is consistent with the previous literature that suggests that the relationship between sociodemographic variables and acute mental health services utilization is complex and varies across studies. While some research indicates that demographic factors are not significantly associated with mental health service utilization [41,42,43], others highlight specific sociodemographic influences [44,45].

However, the study found that educational attainment and housing status initially showed trends towards significant associations with inpatient admissions and ED visits one year post-RASP assessment. Specifically, study participants who indicated having lower education levels or those living in unstable housing were more likely to utilize acute mental health services. This finding aligns with the previous literature connecting lower socioeconomic status and housing instability to increased mental health problems [45,46,47]. However, once the Bonferroni correction was applied, neither educational level nor housing was significantly associated with acute mental health use. This implies that the initial observed relationships between these variables and acute service use may be due to random chance, highlighting the significance of correcting for multiple comparisons to reduce the risk of Type I error. Although our adjusted analysis cautions against interpreting these associations, the observed trends warrant further investigation in larger, powered studies that can use multivariable models to clarify complex causal relationships.

### 4.2. Clinical Significance/Future Implications

The results of this study have significant implications for the delivery and planning of mental health services. The low rate of acute mental health services used by study participants, though with a high mental health symptom burden, implies that early and rapid access intervention models such as RASP may offer the promise of potentially diverting patients away from high-cost acute mental health services. Although no significant associations were found between patient characteristics and service utilization, the descriptive findings remain meaningful, particularly in highlighting patterns that can inform service delivery. Guided by Andersen’s Behavioral Model as a guiding framework, the study reinforces the need to consider not only clinical need but also predisposing and enabling factors when developing and evaluating mental health programs. This justifies the importance of continued funding for rapid access and stabilization interventions as part of transforming global mental health systems. Furthermore, the observed nonstatistical association between housing instability, educational level, and acute service use highlights the need for integrated interventions that address sociodemographic predictors and acute mental health utilization. Adopting targeted early interventions in the mental health delivery system may enhance an effective, equitable care system for these vulnerable populations. Future studies with more extended follow-up periods or matched control groups are warranted to assess long-term outcomes and strengthen causal interpretation. Additionally, results may inform decisions around resource allocation and highlight areas for program refinement.

### 4.3. Limitations

While the study findings offer valuable insights, several limitations should be considered when interpreting the results. This study was conducted within a single provincial program (RASP in Nova Scotia), which may limit the generalizability of findings to other settings with different implementation models, population characteristics, or healthcare system structures. The cross-sectional design used for sociodemographic and clinical profiles without a control group limits the ability to make causal inferences. Longitudinal research or quasi-experimental designs would provide a clearer understanding of how these characteristics influence engagement with acute mental health services. Additionally, although the sample size was adequate for descriptive analyses, the low frequency of outcome events likely limited the study’s power to detect small but potentially meaningful associations. Furthermore, self-reported surveys and administrative data could have introduced reporting bias or misclassification, particularly regarding mental health service use. Again, the study used listwise deletion to handle missing data, given that fewer than 5% of cases were incomplete. While appropriate under the assumption of missing completely at random (MCAR), this approach may have slightly reduced statistical power and introduced bias if the missingness was systematic.

Finally, while the Bonferroni adjustment reduced the risk of Type I errors, it may have increased the likelihood of Type II errors, potentially obscuring real associations. Additionally, due to the low frequency of acute service utilization events and the lack of statistically or nearly significant bivariate associations, multivariable logistic regression was not performed, limiting our ability to assess independent effects and potential confounding among predictors.

## 5. Conclusions

This study provides a comprehensive description of the sociodemographic and clinical profiles of participants who accessed mental health services through the RASP from April 2023 to April 2024, aiming to identify factors associated with the use of acute mental health services twelve months post-RASP enrollment. Although the majority of study participants exhibited high rates of mental health symptoms (anxiety and depression), the overall acute mental health service utilization remained low. Grounded in Andersen’s Behavioral Model of Health Services Use, this study contributes to our understanding of how predisposing characteristics, enabling resources, and perceived or assessed needs shape mental health service use in a publicly funded system. These results suggest that programs like RASP may play a critical role in early intervention and long-term stability of mental health symptoms. While the study initially observed some association trends between participants’ educational attainment, housing status, and acute mental health service utilization, none of these associations remained significant after adjusting for multiple comparisons. Therefore, the study failed to identify associations between sociodemographic and clinical characteristics and acute mental health service use for individuals who accessed RASP one year post-assessment. These results demonstrate the complexity of predicting the association between sociodemographic variables and acute services use and support the requirement for future studies to explore more robust multivariable models to examine the determinants of acute mental health care use.

## Figures and Tables

**Table 1:** Demographic and clinical variables against gender.

Variables		Male N (%)	Female N (%)	Other N (%)	Total N (%)
Age N = 731	<25 yrs	38 (14.9)	94 (20.2)	3 (30.0)	135 (18.5)
	20–40 yrs	96 (37.6)	178 (38.2)	7 (70.0)	281 (38.4)
	41–60 yrs	97 (38.0)	135 (29.0)	0 (0.0)	232 (31.7)
	>60 yrs	24 (9.4)	59 (12.7)	0 (0.0)	83 (11.4)
Sex N = 732	Male	254 (100.0)	0 (0.0)	1 (10.0)	255 (34.8)
	Female	0 (0.0)	468 (100.0)	9 (90.0)	477 (65.2)
Ethnicity N = 721	Indigenous	7 (2.8)	21 (4.5)	0 (0.0)	28 (3.9)
	African	4 (1.6)	17 (3.7)	0 (0.0)	21 (2.9)
	East Asian	3 (1.2)	6 (1.3)	0 (0.0)	9 (1.2)
	Latino	0 (0.0)	2 (0.4)	0 (0.0)	2 (0.3)
	Middle East	6 (2.4)	3 (0.6)	0 (0.0)	9 (1.2)
	South Asian	2 (0.8)	1 (0.2)	0 (0.0)	3 (0.4)
	Caucasian	222 (89.2)	411 (89.0)	9 (1.4)	642 (89.0)
	Other	5 (2.0)	1 (0.2)	1 (10.0)	7 (1.0)
Employment N = 734	Student	17 (6.7)	37 (80.0)	2 (20.0)	56 (7.7)
	Employed	145 (57.3)	260 (56.0)	4 (40.0)	409 (56.3)
	Unemployed	43 (17.0)	74 (15.9)	3 (30.0)	120 (16.5)
	Retired	24 (9.5)	55 (11.9)	0 (0.0)	79 (10.9)
	Other	24 (9.5)	38 (8.2)	1 (10.0)	63 (8.7)
Income range N = 689	No Income	37 (15.4)	45 (10.3)	1 (11.1)	83 (12.1)
	<29,590	53 (22.0)	145 (33.3)	6 (66.7)	204 (29.7)
	29,592–59,180	62 (55.7)	146 (33.5)	2 (22.2)	210 (30.6)
	59,181–93,000	58 (24.1)	78 (17.9)	0 (0.0)	136 (19.8)
	93,001–150,000	24 (10.0)	15 (3.4)	0 (0.0)	39 (5.7)
	>150,000	7 (2.9)	7 (1.6)	0 (0.0)	14 (2.0)
Relationship N = 734	Single	97 (38.3)	171 (36.6)	4 (40.0)	272 (37.3)
	Partner/Married	136 (53.8)	228 (48.8)	5 (50.0)	369 (50.5)
	Separated/divorced	14 (5.5)	55 (11.8)	1 (10.)	70 (9.6)
	Widower	2 (0.8)	10 (2.1)	0 (0.0)	12 (1.6)
	Other	4 (1.6)	3 (0.6)	0 (0.0)	7 (1.0)
Education N = 734	Elementary	4 (1.6)	6 (1.3)	0 (0.0)	10 (1.4)
	High School	73 (28.9)	123 (26.3)	3 (30.0)	199 (27.3)
	Post-secondary	141 (55.7)	296 (63.4)	7 (70.0)	444 (60.8)
	Post-secondary (trade)	28 (11.1)	33 (7.1)	0 (0.0)	61 (8.4)
	Other	7 (2.8)	9 (1.9)	0 (0.0)	16 (2.2)
Housing N = 734	Own house	100 (39.5)	175 (37.5)	1 (10.0)	276 (37.8)
	Rented	89 (35.2)	202 (43.3)	7 (70.0)	298 (40.8)
	With Family	54 (21.3)	85 (18.2)	1 (10.0)	140 (19.2)
	Others	10 (4.0)	5 (1.1)	1 (10.0)	16 (2.2)
GAD-7 N =735	Mild Anxiety	66 (26.1)	89 (19.1)	1 (10.0)	156 (21.4)
	Moderate Anxiety	67 (26.5)	109 (23.4)	4 (40.0)	180 (24.7)
	Severe Anxiety	120 (47.4)	268 (57.5)	5 (50.0)	393 (53.9)
PHQ-9 N = 737	Mild MDD	46 (18.1)	64 (13.7)	1 (10.0)	111 (15.2)
	Moderate MDD	133 (52.4)	219 (46.9)	5 (50.0)	357 (48.8)
	Severe MDD	75 (29.5)	184 (39.4)	4 (40.0)	263 (36.0)
WHO-5 N = 738	Low well-being	34 (13.4)	38 (8.1)	0 (0.0)	72 (9.8)
	High well-being	220 (86.6)	430 (91.9)	10 (100.0)	660 (90.2)
Mobile crisis calls 12 months post-assessment N = 733	Yes	3 (1.2)	4 (0.9)	0 (0.0)	7 (1.0)
	No	252 (98.8)	464 (99.1)	10 (100.0)	726 (99.0)
ED visits 12 months post-assessment N = 733	Yes	18 (7.1)	35 (7.5)	0 (0.0)	53 (7.2)
	No	237 (92.9)	433 (92.5)	10 (100.0)	680 (92.8)
Admitted to inpatient unit 12 months post-assessment N = 733	Yes	4 (1.6)	2 (0.4)	0 (0.0)	6 (0.8)
	No	251 (98.4)	466 (99.6)	10 (100.0)	727 (99.2)

**Table 2:** Sociodemographic and clinical factors associated with acute mental health services utilization of RASP participants one year post-assessment.

Variables		Mobile Crisis Post-Assessment					ED Visits Post-Assessment					Inpatient Admission Post-Assessment				
		YES N (%)	NO n (%)	X^2^/Fisher Exact	*p*-Value	Phil/ Cramer’s V	YES n (%)	NO n (%)	X^2^/Fisher Exact	*p*-Value	Phil/ Cramer’s V	YES n (%)	NO n (%)	X^2^/Fisher Exact	*p*-Value	Phil/ Cramer’s V
Age N = 734	<25 yrs	1 (0.7)	135 (99.3)	4.56	0.16	0.08	12 (8.8)	124 (91.2)	3.95 *	0.23	0.07	2 (1.5)	134 (98.5)	1.70	0.57	0.05
	20–40 yrs	5 (1.8)	276 (98.2)				23 (8.2)	258 (91.8)				3 (1.1)	278 (98.9)			
	41–60 yrs	0 (0.0)	232 (100.0)				16 (6.9)	216 (93.1)				1 (0.4)	231 (99.6)			
	>60 yrs	1 (1.2)	84 (98.8)				2 (2.4)	83 (97.6)				0 (0.0)	85 (100.0)			
Gender N = 733	Male	3 (1.2)	252 (98.8)	1.06	0.73	0.02	18 (7.1)	237 (92.9)	0.83 *	0.71	0.03	4 (1.6)	251 (98.4)	3.40	0.26	0.06
	Female	4 (0.9)	464 (99.1)				35 (7.5)	433 (92.5)				2 (0.4)	466 (99.6)			
	Other	0 (0.0)	10 (100.0)				0 (0.0)	10 (100.0)				0 (0.0)	10 (100.0)			
Sex N = 736	Male	3 (1.2)	255 (98.8)		0.70	−0.02	18 (7.0)	240 (93.0)	0.03 *	0.88	0.01	4 (1.6)	254 (98.4)		0.19	−0.06
	Female	4 (0.8)	474 (99.2)				35 (7.3)	443 (92.7)				2 (0.4)	476 (99.6)			
Ethnicity N = 724	Indigenous	0 (0.0)	29 (100.0)	9.83	0.42	0.07	1 (3.4)	28 (96.6)	4.02	0.67	0.09	0 (.0)	29 (100.0)	7.73	1.00	0.03
	African	1 (4.8)	20 (95.2)				1 (4.8)	20 (95.2)				0 (0.0)	21 (100.0)			
	East Asian	0 (0.0)	9 (100.0)				0 (0.0)	9 (100.0)				0 (0.0)	9 (100.0)			
	Latino	0 (0.0)	2 (100.0)				0 (0.0)	2 (100.0)				0 (0.0)	2 (100.0)			
	Middle East	0 (0.0)	9 (100.0)				2 (22.2)	7 (77.8)				0 (0.0)	9 (100.0)			
	South Asian	0 (0.0)	3 (100.0)				0 (0.0)	3 (100.0)				0 (0.0)	3 (100.0)			
	Caucasian	6 (0.9)	638 (99.1)				49 (7.6)	595 (92.4)				6 (0.9)	638 (99.1)			
	Other	0 (0.0)	7 (100.0)				0 (0.0)	7 (100.0)				0 (0.0)	7 (100.0)			
Employment N = 731	Student	0 (0.0)	57 (100.0)	0.84	1.00	0.05	3 (5.3)	54 (94.7)	7.51 *	0.11	0.10	0 (0.0)	57 (100.0)	0.91	1.00	0.06
	Employed	5 (1.2)	405 (98.8)				38 (9.3)	372 (90.7)				5 (1.2)	405 (98.8)			
	Unemployed	1 (0.8)	120 (99.2)				7 (5.8)	114 (94.2)				1 (0.8)	120 (99.2)			
	Retired	1 (1.3)	78 (98.7)				1 (1.3)	78 (98.7)				0 (0.0)	79 (100.0)			
	Other	0 (0.0)	64 (100.0)				4 (6.3)	60 (93.8)				0 (0.0)	64 (100.0)			
Income Range N = 689	No Income	1 (1.2)	82 (98.8)	3.23	0.60	0.07	4 (4.8)	79 (95.2)	5.77 *	0.32	0.09	0 (0.0)	83 (100.0)	3.13	0.67	0.08
	<29,590	2 (1.0)	204 (99.0)				12 (5.8)	194 (94.2)				3 (1.5)	203 (98.5)			
	29,592–59,180	4 (1.9)	206 (98.1)				21 (10.0)	189 (90.0)				3 (1.4)	287 (98.6)			
	29,181–93,000	0 (0.0)	136 (100.0)				13 (9.6)	123 (90.4)				0 (0.0)	136 (100.0)			
	93,001–150,000	0 (0.0)	39 (100.0)				1 (2.6)	38 (97.4)				0 (0.0)	39 (100.0)			
	>150,000	0 (0.0)	15 (100.0)				1 (6.7)	14 (93.3)				0 (0.0)	15 (100.0)			
Relationship N = 734	Single	1 (0.4)	272 (99.6)	3.68	0.52	0.52	19 (7.0)	254 (93.0)	1.02	0.92	0.04	3 (1.1)	270 (98.9)	3.45	0.63	0.04
	Partner/Married	5 (1.3)	367 (98.7)				29 (7.8)	343 (92.2)				2 (0.5)	370 (99.5)			
	Separated/Divorced	1 (1.4)	69 (98.6)				4 (5.7)	66 (94.3)				1 (1.4)	69 (98.6)			
	Widower	0 (0.0)	12 (100.0)				1 (8.3)	11 (91.7)				0 (0.0)	12 (100.0)			
	Other	0 (0.0)	7 (100.0)				0 (0.0)	7 (100.0)				0 (0.0)	7 (100.0)			
Education N = 734	Elementary	0 (0.0)	10 (100.0)	4.47	0.35	0.08	1 (10.0)	9 (90.0)	9.11	0.04	0.12	0 (0.0)	10 (100.0)	10.79	0.02	0.14
	High School	1 (0.5)	200 (99.5)				8 (4.0)	193 (96.0)				2 (1.0)	199 (99.0)			
	Post-secondary	4 (0.9)	441 (99.1)				35 (7.9)	410 (92.1)				1 (0.2)	444 (99.8)			
	Post-secondary (trade)	2 (3.2)	60 (96.8)				9 (14.5)	53 (85.5)				3 (4.8)	59 (95.2)			
	Other	0 (0.0)	16 (100.0)				0 (0.0)	16 (100)				0 (0.0)	16 (100)			
Housing N = 734	Own house	1 (0.4)	276 (99.6)	7.11	0.05	0.11	21 (7.6)	256 (92.4)	0.86 *	0.85	0.03	1 (0.4)	276 (99.6)	10.32	0.01	0.19
	Rented	5 (1.7)	295 (98.3)				20 (6.7)	280 (93.3)				2 (0.7)	298 (99.3)			
	With Family	0 (0.0)	141 (100.0)				10 (7.1)	131 (92.9)				1 (0.7)	140 (99.3)			
	Others	1 (6.3)	15 (93.8)				2 (12.5)	14 (87.5)				2 (12.5)	14 (87.5)			
GAD-7 N = 735	Mild Anxiety	1 (0.6)	160 (99.4)	0.33	1.00	0.02	8 (5.0)	153 (95.0)	2.09 *	0.35	0.05	1 (0.6)	160 (99.4)	2.01	0.36	0.06
	Moderate Anxiety	2 (1.1)	178 (98.9)				12 (6.7)	168 (93.3)				0 (0.0)	180 (100)			
	Severe Anxiety	4 (1.0)	390 (99.0)				33 (8.4)	361 (91.6)				5 (1.3)	389 (98.7)			
PHQ-9 N = 737	Mild MDD	0 (0.0)	114 (100.0)	0.87	0.43	0.04	6 (5.3)	108 (94.7)	0.85 *	0.65	0.03	0 (0.0)	114 (100.0)	0.85	0.75	0.04
	Moderate MDD	4 (1.1)	354 (98.9)				28 (7.8)	330 (92.2)				3 (0.8)	355 (99.2)			
	Severe MDD	3 (1.1)	326 (98.9)				19 (7.2)	246 (92.8)				3 (1.1)	262 (98.9)			
WHO-5 N = 738	Low Well-being	0 (0.0)	74 (100.0)	0.79 *	0.63	0.03	4 (5.4)	70 (94.6)	0.39 *	0.64	0.02	0 (0.0)	74 (100.0)	0.67 *	0.64	0.03
	High Well-being	7 (1.1)	657 (98.9)				49 (7.4)	615 (92.6)				6 (0.9)	658 (99.1)			

* = chi-square.

## Data Availability

The datasets used and/or analyzed during the current study are available from the corresponding author upon reasonable request.

## Abbreviations

RASP	Rapid Access and Stabilization Program
PHP	Primary Healthcare Providers
ED	Emergency Department
WHO	World Health Organization
CIHI	Canadian Institute for Health Information
NSHA	Nova Scotia Health Authority
QEII	Queen Elizabeth II
CZ	Central Zone
SPSS	Statistical Package for Social Sciences
MDD	Major Depressive Disorder
GAD	Generalized Anxiety Disorder
PHQ	Patient Health Questionnaire
WHO-5	World Health Organization 5 Well-being Index
